# Analysis of Induced Field in the Brain Tissue by Transcranial Magnetic Stimulation Using Halo-V Assembly Coil

**DOI:** 10.1155/2022/7424564

**Published:** 2022-07-14

**Authors:** Khaleda Akhter Sathi, Md. Kamal Hosain, Md. Azad Hossain

**Affiliations:** ^1^Department of Electronics & Telecommunication Engineering, Chittagong University of Engineering & Technology, Chittagong 4349, Bangladesh; ^2^Department of Electronics & Telecommunication Engineering, Rajshahi University of Engineering & Technology, Rajshahi 6204, Bangladesh

## Abstract

As a noninvasive neuromodulation technique, transcranial magnetic stimulation (TMS) has already exhibited a great impact in clinical application and scientific research. This study presents a finite element method-based simulation of the Halo-V assembly (HVA) coil placed on the five-shell spherical human head model to examine the distributions of induced electric and magnetic fields. The performance of the designed HVA coil is evaluated by comparing the simulation results with the commercially available Halo-FO8 (HFA) assembly coil and standard single coils including the Halo and V coils. The simulation results indicate that the HVA coil shows an improved focality in terms of electric field distribution than the other single and assembly stimulation coils. Additionally, the effects of a magnetic shield plate and magnetic core on the designed HVA coil are investigated. Results indicate that the magnetic shield plate and magnetic core are proficient in further improving the stimulation focality. Therefore, the HVA TMS coil results in a safe and effective stimulation with enhanced focality of the target region as compared to the existing assembly coil.

## 1. Introduction

Transcranial magnetic stimulation (TMS) has shown a greater therapeutic outcome for some neural conditions such as major depressive disorder, traumatic brain injury, Parkinson's disease, and posttraumatic stress disorder [1–4]. This stimulation technique requires a magnetic coil normally placed on the subject's head that is fed with a high-valued short-duration current pulse generator [5]. The electric current conveying coil produces a magnetic field that results in an electric field inside the brain tissue medium [6]. A localized axial depolarization is produced by the induced electric field in the underlying cortical tissue which has a therapeutic advantage for neural disorders [7]. To ensure a greater therapeutic effect, the induced electric field should have to be strong enough, so that it can depolarize the targeted neurons that are responsible for the neural disorders [8, 9]. Moreover, the strong electric field intensity should have to be induced in a precise location called focality that ensures target neural tissue excitations. Thus, the induced field intensity and focality are the two main concerns to providing safe and effective TMS treatment. The geometrical structure of the stimulation coil has the main impact on the stimulation intensity as well as focality.

Therefore, many efforts have been made in the development of a new TMS coil configuration in the past two decades [10–12]. For instance, the standard FO8 coil comprising two contiguous circular loops with opposing current flow is developed and used in the early stage [13]. This coil creates a relatively focal electric field with maximum intensity under the center of the structure where the two loops meet [14]. However, this coil configuration is not appropriate for directly stimulating the deep neurons due to the rapid decrease of the induced electric field with the increase of the distance from the vertex of the head. On the other hand, the development of a Halo coil consisting of a single large circular coil placed around the head led to a deeper stimulation of the brain region [15, 16]. Besides this deeper stimulation of the brain region, the Halo coil induces a highly intense electric field that results in overstimulation of the target neurons. Moreover, the Halo coil has less focality. Therefore, the HFA coil [17], i.e., the Halo coil working with a standard FO8 coil is developed to meet the requirements of desired electric field intensity at a larger depth region. However, this coil has a limitation in terms of focality that causes an overstimulation of the scalp region as well as the deeper region of the brain [18]. As a result, the side effects including the risk of pain and seizure may be caused [19].

Hence, a novel assembly coil can reduce therapeutic side effects as compared to the existing commercial coils by improving the stimulation depth as well as the focality. For this reason, this study introduces an assembly coil named HVA as a neurostimulating coil that can limit the induced field within a lesser region of the scalp as well as the cortical region to enhance the stimulation focality. The distributions of induced magnetic and electric fields in the five-shell spherical-shaped head model are comprehensively analyzed based on the finite element method (FEM). Moreover, depending on the simulation results, a comparison between the existing assembly coil and the proposed assembly coil is performed to evaluate the coil performance.

The organization of the study is as follows: Section 1 presents the introduction of the study. A detailed explanation of the coil modeling and simulation methods is described in Section 2. In Section 3, the simulation results are described with their analysis. The performance evaluation of the designed coil is conducted in 4. Finally, Section 5 shows concluding remarks.

## 2. Materials and Methods

### 2.1. Five-Shell Head Model

**Figure 1:**
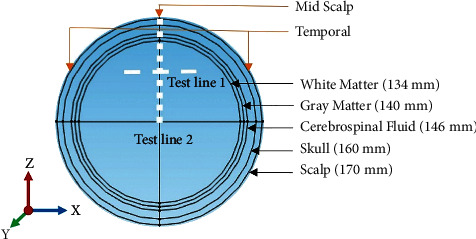
The cross-sectional view of five layers of the spherical human head model.

In this simulation, a spherical five layers model is employed to represent the human head [20].  Figure 1 shows the cross-section of the modeled human head that is comprised of five different anatomical layers including the scalp, skull, cerebrospinal fluid, gray matter, and white matter, respectively. All layers are indicated in the inset of the three-dimensional head model of the Cartesian coordinates system. The outer and inner diameters of scalp structure are 170 mm and 160 mm, respectively, while the most inner tissue, i.e., the white matter region is modeled with a diameter of 134 mm. The thickness of four anatomical layers of scalp, skull, cerebrospinal fluid, and gray matter is 10 mm, 14 mm, 6 mm, and 6 mm, respectively [20]. The inner portion of the gray matter is considered as the white matter. The working frequency of the stimulation current is generally between 2500 and 5000 Hz, but the common value of 2500 Hz is adopted in this study. The electromagnetic properties of the different tissues of the head model at an operating frequency of 2500 Hz are given in Table 1 [21]. However, the thickness of anatomical layers and values of conductivity of tissues may vary in different phantom models reported in the literature. For the actual human head, the anatomical layers will be inhomogeneous and their properties will vary from the reported values in a phantom model. Hence, the field distribution for the actual head will be slightly different from the phantom model, which is considered as the limitation of this work.

### 2.2. Coil Geometry and Excitation

**Figure 2:**
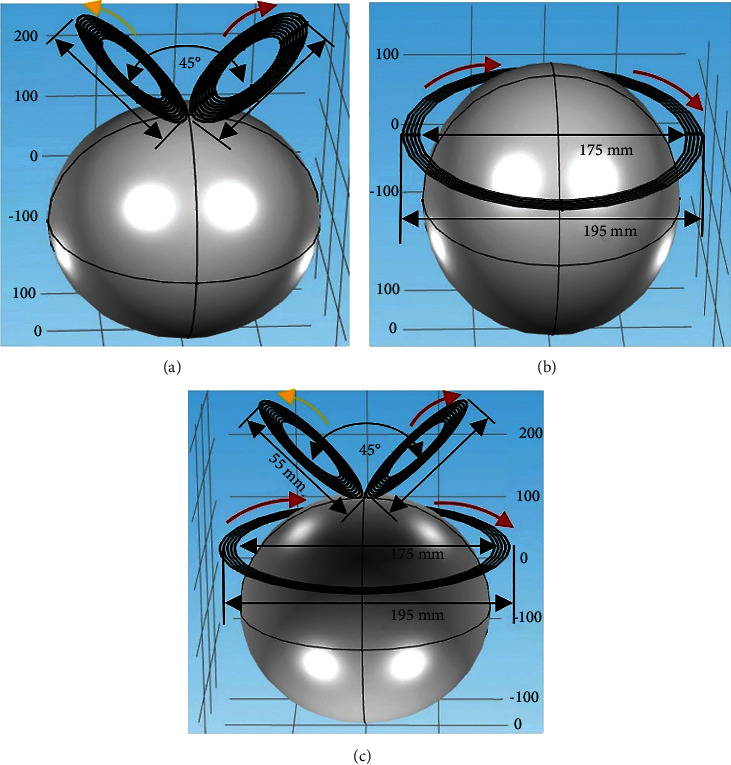
Geometrical structure of coils. (a) V coil. (b) Halo coil. (c) HVA coil. The red arrow line indicates the clockwise current direction and the yellow arrow line indicates the anticlockwise current direction.

Figure 2 shows the geometrical structure of the three coils named V, Halo, and HVA, respectively. The V coil has the same dimension as the conventional FO8 coil with inner and outer diameters of 55 mm and 95 mm, respectively, as shown in Figure 2(a). The designed V coil consists of nine turns. The two wings of the V coil are separated by an angle of 45° which is located 5 mm above the midscalp of the head. The current pulse in two wings of the coil is set to flow in opposite directions. On the other hand, Figure 2(b) shows the Halo coil with a dimension of 175 mm and 195 mm for inner and outer diameters, respectively. The Halo coil is positioned 90 mm below the midscalp of the head. The direction of current flow in the Halo coil is similar to one of the wings of the V coil and opposite to the other wing. The HVA coil configuration, as shown in Figure 2(c), consists of two coils: a Halo coil and a V coil. These two coils have a different number of turns: 9 for both wings of the V coil and 5 for the Halo coil which makes a total of 23 turns. The one part of the HVA coil, i.e., V coil is placed at a distance of 5 mm from the midscalp of the head model. The position of the V coil is set at this position to reduce unwanted tissue damage. Similarly, the other part, i.e., the Halo coil is placed at 90 mm from the midscalp of the head model. The design parameters of the three coils are given in Table 2. Each of the coils is modeled by considering the torus shape of copper material with an electrical conductivity of 5.8 × 10^7^ S/m. Also, the coils are fed with a current pulse of amplitude 5000 A and a frequency of 2500 Hz [21].

### 2.3. Governing Equation and Meshing

The generation of fields by feeding a high amplitude current pulse to the coil follows Maxwell's fourth law of ampere's circuit law. Where the distribution of the charge carrier in the closed-loop coil generates a magnetic field that is in a direction perpendicular to the coil surface, as a result, the changing magnetic field induces an electric field in the conductive head tissue medium. The differential form of the following equations (1)–(4) is used to represent these scenarios, where *J* and *J*_*e*_ represent the current density vector and the externally generated current density, respectively. The magnetic field vector and potential are indicated by H and A, whereas B is the magnetic intensity vector. Moreover, the induced electric field intensity and displacement vector are denoted as *E* and *D*, respectively.

(1) $$\begin{array}{c} J=\nabla \times H=\sigma E+j\omega \;D+J_{e}, \end{array}$$

(2) $$\begin{array}{c} E=-\nabla V-j\omega A, \end{array}$$

(3) $$\begin{array}{c} B=\nabla \times A, \end{array}$$

(4) $$\begin{array}{c} D=\varepsilon_{0}\varepsilon_{r}E. \end{array}$$

Based on the above equations, the coils are simulated in COMSOL Multiphysics 5.0a software for frequency domain analysis of the electromagnetic field. The magnetic and electric field (mef) interface from the AC/DC module of the COMSOL software is used for simulation. In simulation, the total model domain including head and coil geometries are divided into several subdomains for solving the governing equations [22]. Since the dimension of the coil is small as compared to the head model, the mesh elements for the coil geometries are set to denser than the head tissue medium to compute the actual changes of the electric field in the conductive head tissue medium. By considering the computational time and memory requirement, the fine tetrahedral meshing elements are considered with sizes ranging from 8 mm to 100 mm. However, a smaller mesh size will increase numerical accuracy. Moreover, the maximum element growth rate and curvature factor are set at the value of 5 and 0.9, respectively. The complete mesh consists of the domain, boundary, and edge element with numbers 139062, 40104, and 9232, respectively.

## 3. Results and Analysis

**Figure 3:**
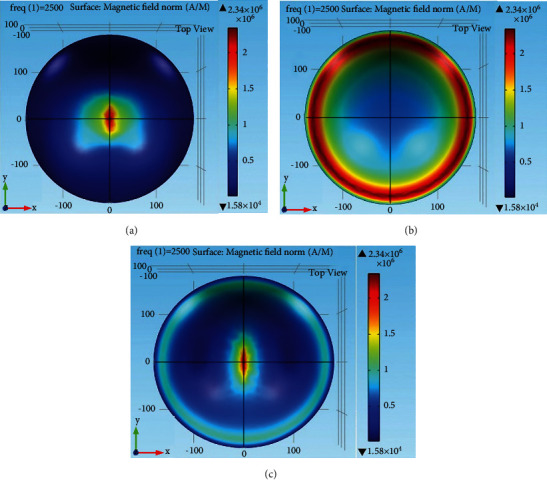
Magnetic field distribution of coils. (a) V coil, (b) Halo coil, and (c) HVA coil.

The designed coils are simulated with COMSOL Multiphysics 5.0a software. In the xy-plane, the surface distributions of H-field for V, Halo, and HVA coils at the head tissue medium are shown in Figure 3. From Figure 3(a), it is shown that the magnetic field intensity of the V coil is greater at the center of the midscalp region because the two coil wings meet in that region. On the contrary, the midscalp region is free from the magnetic field induced by the Halo coil as shown in Figure 3(b). It produces the H-field at the temporal region of the head model. The magnetic field of the HVA coil as shown in Figure 3(c) is concentrated at the lesser region of the midscalp as compared to the V coil. Moreover, it has a reduced H-field at the temporal region as compared to the Halo coil. Therefore, the concentrated H-field with lesser area results in reducing the area of the induced electric field which is responsible for the activation of unwanted head tissues.

**Figure 4:**
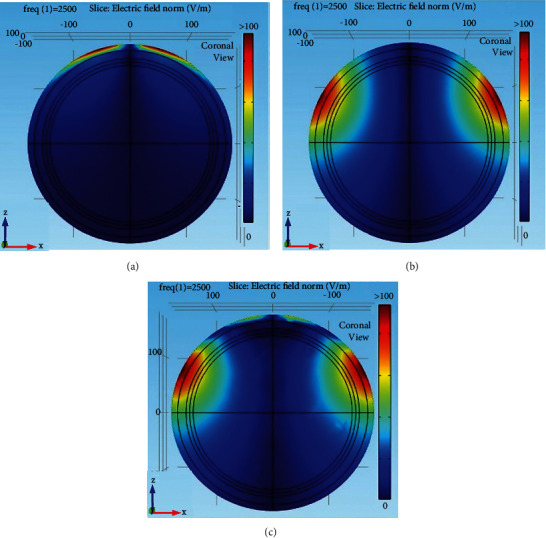
Electric field distribution of coils. (a) V coil, (b) Halo coil, and (c) HVA coil.

The slice views of electric field distribution for V, Halo, and HVA coils in the zx-plane are shown in Figure 4. From Figure 4(a), it is observed that the total maximum electric field value of the V coil is 312 V/m with a lower depth of stimulation in the midscalp region. On the other hand, the Halo coil induces a total maximum electric field of 550 V/m with a greater field penetration depth at the inner region of the white matter on both left and right temporal (Figure 4(b)). This electric field is also spread over a wide region. The total maximum electric field value of the HVA coil as shown in Figure 4(c) is found to be 482 V/m. It shows the similar field penetration depth of the Halo coil but reduces the area of stimulation at the scalp on the temporal. Moreover, the electric field value is under the threshold in the cortex region on the midscalp of the head. Therefore, compared with the V coil, it has greater field penetration depth and has a lower area of stimulation as compared to the Halo coil.

**Figure 5:**
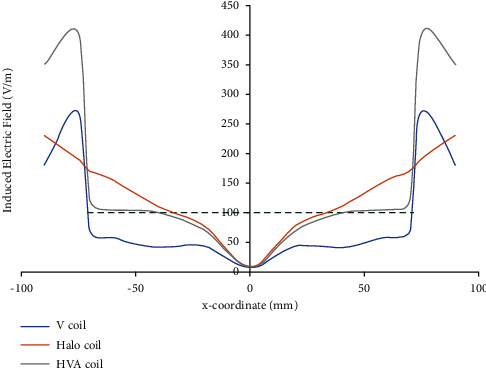
Electric field distribution along test line 1. Green dotted line indicates the neuron stimulation threshold.

The line graphs of the electric field by V, Halo, and HVA coils along the test line 1 (Figure 1) are shown in Figure 5. The test line 1 is considered parallel to the *x*-axis with endpoints of (0, −80, 80) mm and (80, −80, 80) mm where the temporal region of the head model is located. The electric field induced by the V coil reaches the threshold value (>100 V/m) [23] at both endpoints of (>70 mm and < −70 mm) of the test line that representing lesser field penetration depth. On the contrary, the threshold electric field intensity produced by the Halo coil can stimulate the deeper region of (>25 mm and <−25 mm) along the test line. But the electric field curve is linearly decreased from both endpoints towards the center of the spherical head model indicating lower focality. In the case of HVA coil, the threshold electric field intensity is found at depth (>30 mm and <−30 mm). Thus, compared to V and Halo coils, the HVA coil improves the penetration depth as well as focality within the range of −90 mm to −30 mm and 30 mm to 90 mm, respectively.

**Figure 6:**
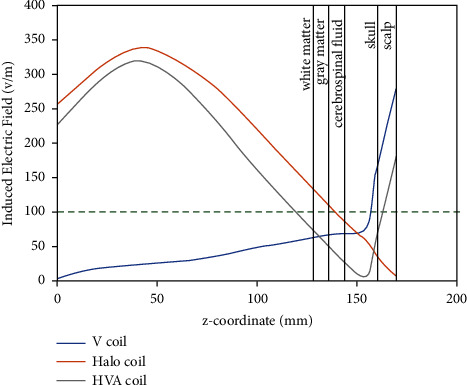
Electric field distribution along test line 2. Green dotted line indicates the neuron stimulation threshold.

Moreover, Figure 6 shows the line graph of the induced electric field for V, Halo, and HVA coils along test line 2 (Figure 1). The test line 2 is considered along the *z*-axis with endpoints of (0, 0, 0) mm and (0, 0, 170) mm, where the midscalp of the head model is located. The induced electric intensity of the V coil is higher than the threshold only at the skull surface from 155 mm to 170 mm on the midscalp of the head and produces an under threshold electric field intensity at the inner region of the skull below 155 mm. On the contrary, an electric field over threshold is induced by the Halo coil at the region below 150 mm. Hence, the skull surface of the midscalp is free from stimulation. For the HVA coil, the over threshold electric field intensity is induced at the skull surface from 155 mm to 170 mm as well as at the deeper region below 120 mm from top of the head. Therefore, the inner region of the white matter is stimulated by an HVA coil with a threshold electric field that indicates a suitable deeper penetration.

### 3.1. Effect of Magnetic Core

**Figure 7:**
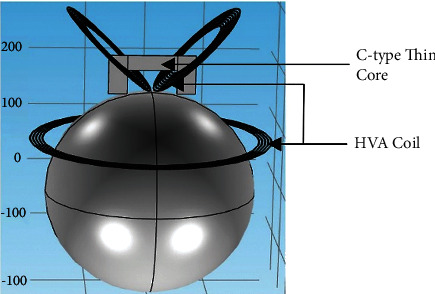
The HVA coil with C-type thin core.

**Figure 8:**
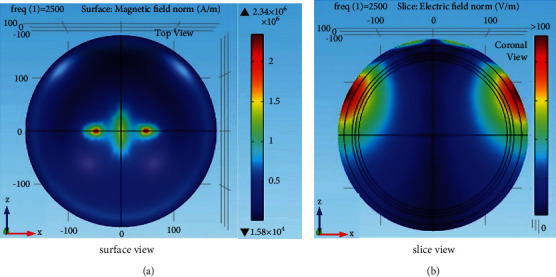
(a) Magnetic field and (b) electric field distribution of the HVA coil with C-type thin core.

To improve the stimulation effect such as electric field intensity and focality, the magnetic core can be attached to the designed coil. However, considering the side effects such as heat energy and large size [24], the C-type core with thinner dimensions is selected with a depth of 1 mm, a width of 80 mm, and a height of 35 mm. The nanocrystalline alloy material is used as the core material that has high saturated flux density and curie temperature at a lower frequency of below 100 kHz [25]. The core is placed at the center of the V coil as shown in Figure 7. The Metglas nanocrystalline material has a conductivity of 0.833 MS/m, a relative permittivity of 1, and a relative magnetic permeability of 1100 [24]. After completing the simulation with a magnetic core, the results are analyzed. In Figure 8(a), it is shown that the magnetic field for the HVA coil with the core is more concentrated with large field intensity than the coil without the magnetic core shown in Figure 3(c). The improvement of the field intensity value is because of passing the magnetic induction lines in the high permeable core regions rather than the air gap. Moreover, the increment of the magnetic field intensity results in increasing the electric field intensity by 0.2% with reducing the area of stimulation (focality) as shown in Figure 8(b).

### 3.2. Effect of Magnetic Shield

**Figure 9:**
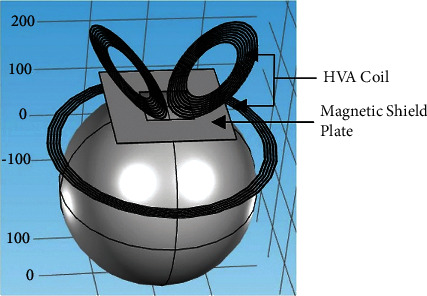
The HVA coil with a magnetic shield plate.

**Figure 10:**
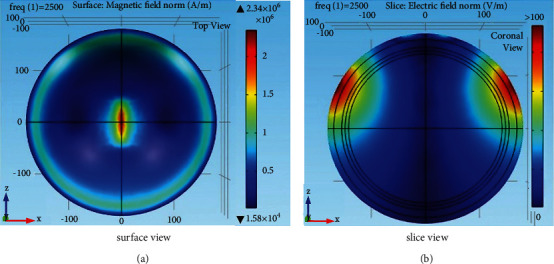
(a) Magnetic field and (b) electric field distribution of the HVA coil with a magnetic shield plate.

In order to improve the focusing of the electric field distribution [26], the magnetic shield plate is placed on the designed HVA coil. A conductor plate is placed under the V coil with a conductivity of 1.12 × 10^7^ S/m and a thickness of 1 mm. The outer length and width of the rectangular shield plate are 200 mm and 200 mm as shown in Figure 9. There is a hole in the middle of the conductor plate. The length and width of the hole are set as 80 mm and 80 mm, respectively, allowing the magnetic induction lines to induce an electric field in the neural tissue medium. On the other hand, the solid magnetic shield area blocks the magnetic induction lines to pass through them.  Figure 10 shows the induced magnetic and electric fields by the HVA coil with the composition of the magnetic shield. It shows that the area of stimulation shown in Figure 10(a) is reduced as compared to the HVA coil without a magnetic shield. The volume of stimulation where the number of an electric field is greater than the threshold is found as 4.61E-04 m^3^ and 4.52E-04 m^3^ for HVA without and with a shield, respectively. Thus, it represents improved focusing than the HVA coil without the conductive shield. Moreover, the value of the electric field is increased by 3% (Figure 10(b)) as compared to the designed HVA coil without a core and shield as shown in Figure 4(c).

## 4. Performance Evaluation

**Figure 11:**
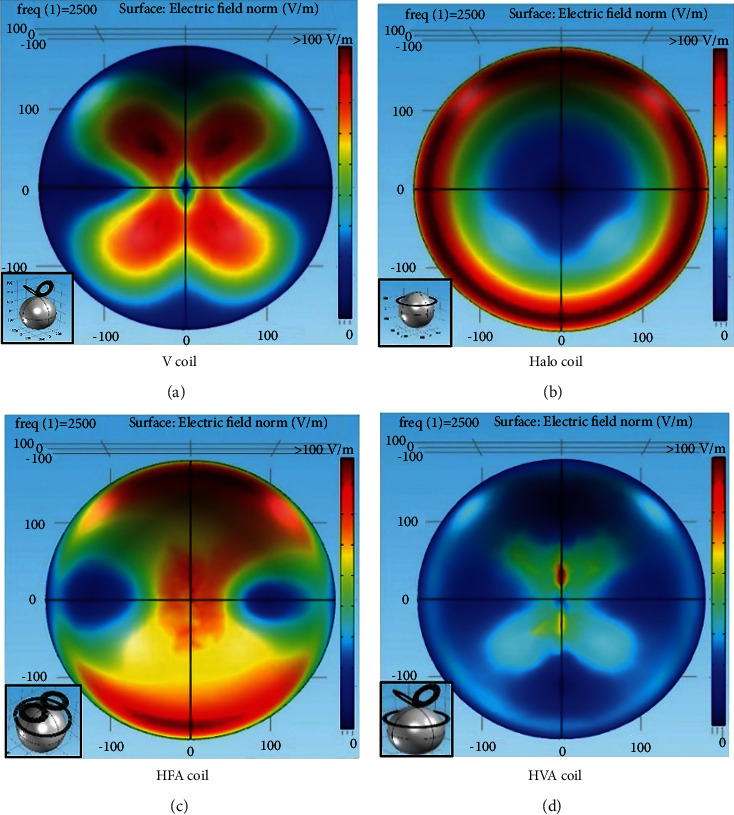
Comparison of the surface electric field distribution of different coils.

**Figure 12:**
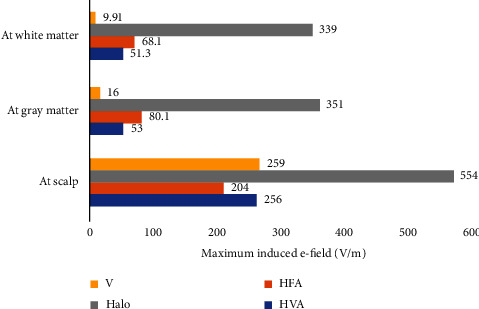
Comparison of the maximum induced electric field on the scalp, gray matter, and white matter of the head model for the V, Halo, HFA, and HVA coils.

To evaluate the performance of the designed HVA coil, its induced electric field predicted in the spherical model is compared with the existing single and assembly coils in terms of the area of stimulation. The distribution of induced electric field on the surface of the scalp for four different coils, i.e., V, Halo, HFA, and HVA is shown in Figure 11. From the results of electric field distribution, it is clear that the stimulation area of the HVA coil is lower than the other single and assembly coils reported here. Hence, HVA coil can reduce the undesired tissue excitation in the scalp region than the V, Halo, and HFA coils. Moreover, the bar plot of the maximum electric field induced by four different coils on the white matter, gray matter, and scalp region of the head model is shown in Figure 12. Results show that the HVA coil stimulates the white matter region with weaker intensities than the HFA coil. However, the comparable ratios of the induced electric field in the scalp and the white matter region for both the HVA and HFA coils are found as 4.99 and 2.99, respectively. In case of scalp to gray matter intensity ratio, the values are found as 4.83 and 2.54 for HVA and HFA, respectively. These indicate that the HVA coil can reduce the overstimulation of neurons near the stimulation site.

**Figure 13:**
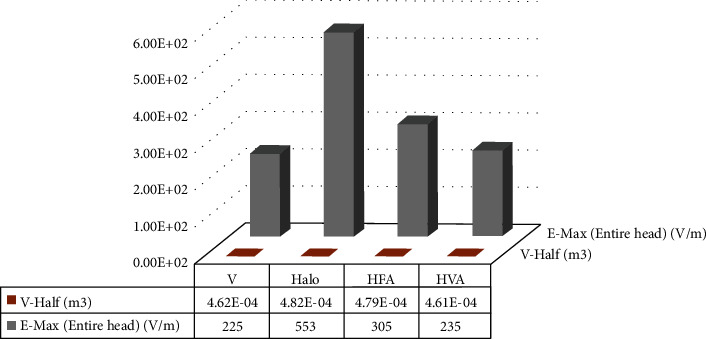
Focality measurement of single and assembly coils in terms of V-half and maximum electric field.

Furthermore, the performance of the HVA coil in terms of focality is also evaluated to ensure the probability of stimulating the targeted neurons. The focality, *S*_1/2_ calculation is performed by using the following equation [24]:

(5) $$\begin{array}{c} S_{1/2}=\frac{V_{1/2}}{D_{1/2}}, \end{array}$$

where *V*_1/2_ (V-half) represents the volume within which the electric field is greater than half of E-Max, and *D*_1/2_ is the distance from the midscalp to the white matter. For both single and assembly coils, the values of V-half and E-Max are presented as bar plots in Figure 13. From the values of the V-half, it can be shown that the HVA coil has a lower volume of stimulation over three coils. Therefore, the reduced volume of stimulation reduces the value of *S*_1/2_ , which results in an increased focusing performance of the HVA coil.

## 5. Conclusion

In conclusion, a new HVA TMS coil consisting of traditional Halo and V coils is designed in this study. The effect of the HVA coil on the ball-shaped human head model is investigated by analyzing the distribution of the induced magnetic field and electric field. Moreover, the impacts of the magnetic core and shield plate on the improvement of the induced electric field intensity as well as focality are evaluated by employing it in the HVA coil. Also, the performance of the HVA coil is evaluated by comparing its simulation results with the commercially available assembly and single coils. The simulation results show that the HVA coil shows an improved focality in terms of electric field distribution by reducing the surface area of stimulation on the scalp as compared to other reported stimulation coils. Hence, the proposed HVA is suitable for stimulating precise target areas for the treatment of a specific disease and reducing unwanted tissue damage and side effects. This work also has some limitations. For instance, the conductivity of the scalp used in this work should be more than the skull region to find out more realistic results. Besides this, the mesh size needs to be smaller than the chosen values to get more accurate results. Thus, the performance of the proposed HVA coil with a more realistic head model can be determined in future to meet the actual clinical desire in terms of depth and focality. For clinical application, the physician can use this coil for TMS therapy for effective treatment of new neurological diseases as they are continually trying to understand the variation of brain tissue stimulation under novel coil design. However, rigorous in vitro testing and in vivo testing are required before clinical application.

## Figures and Tables

**Table 1:** Electromagnetic properties of the five different anatomical layers at an operating frequency of 2500 Hz [21].

Tissue name	Electrical conductivity (S/m)	Relative permittivity	Relative permeability
Scalp	0.0002	1140	0.99
Skull	0.0203	1440	1.00
Cerebrospinal fluid	2	109	0.99
Gray matter	0.104	78100	0.99
White matter	0.0645	34300	0.99

**Table 2:** Design parameters of three coils.

Coil name	Inner diameter **d**_**in**_ (mm)	Outer diameter **d**_**out**_ (mm)	Total coil turns	Angle between two wings *θ* (degree)
V	55	95	18	45°
Halo	175	195	5	—
HVA	V = 55	V = 95	23	45°
	Halo = 175	Halo = 195		

## Data Availability

The data used to support the findings of this study are included within the article.
